# Dynamics and challenges of technology transfer in Colombia: a systematic literature review

**DOI:** 10.3389/frma.2025.1628141

**Published:** 2025-09-10

**Authors:** Alejandro Valencia-Arias, Karla Cristina Bonilla Restrepo, Eliana Villa-Enciso, Jackeline Valencia, Juan Camilo Rua Hernandez, Diana Marleny Ramírez-Ramírez

**Affiliations:** ^1^Facultad de Ciencias Económicas y Administrativas, Instituto Tecnológico Metropolitano, Medellín, Colombia; ^2^Coordinación de Investigación e Innovación, Fundación Universitaria Católica del Norte, Medellín, Colombia; ^3^Facultad de Artes y Humanidades, Instituto Tecnológico Metropolitano, Medellín, Colombia; ^4^Vicerrectoría de Investigación y Postgrado, Universidad de Los Lagos, Osorno, Chile; ^5^Departamento de Finanzas, Instituto Tecnológico Metropolitano, Medellín, Colombia

**Keywords:** technology transfer, innovation, university-business collaboration, public policies, innovation system

## Abstract

**Introduction:**

Technology transfer in Colombia plays a critical role in promoting innovation and regional development. However, its implementation continues to face significant institutional, economic, and cultural challenges that hinder its effectiveness and sustainability.

**Methods:**

A systematic literature review was conducted in accordance with the PRISMA 2020 guidelines. The databases Scopus and Web of Science were used to identify peer-reviewed articles that addressed knowledge and technology transfer within the Colombian context.

**Results:**

The analysis revealed multiple barriers: institutional fragmentation, inadequate investment in R&D, lack of incentives for collaboration, and weak trust between academia and industry. Additionally, organizational rigidity and the limited adaptation of international transfer models to local contexts were identified as factors that reduce the effectiveness of these processes.

**Discussion:**

The findings highlight the need to acknowledge regional particularities, foster inclusive governance, and promote horizontal relationships among stakeholders. Effective international strategies, such as open innovation, co-creation models, and hybrid technology transfer offices, could be adapted to the Colombian context. Strengthening institutional capacities, increasing R&D funding, and aligning innovation with social and environmental objectives are imperative to build a more robust and inclusive innovation ecosystem.

## Introduction

Technology transfer is defined as the process of transferring knowledge, innovations, and scientific developments that have been generated in academic institutions, research centers, and private enterprises to the manufacturing sector for practical application. This mechanism plays a crucial role in economic development by driving innovation, strengthening business competitiveness, and contributing to the sustainability of long-term growth (Donneys González and Blanco Campins, 2016). In a global environment characterized by the knowledge economy, a country's capacity to adopt and apply new technologies determines its position in international markets and its ability to respond to contemporary productive and social challenges.

In Colombia, technology transfer has been promoted through various strategies and policies formulated by the Ministry of Science, Technology, and Innovation (MinCiencias), in collaboration with other government entities and stakeholders in the innovation system. A number of initiatives have been implemented, including the establishment of research results transfer offices (OTRIs), technology parks, and incentive programs designed to enhance collaboration between academia and the productive sector. Nevertheless, despite these efforts, limitations in the coordination between the actors involved persist, which reduces the effectiveness of these policies (Rivero et al., 2016).

The Colombian context is confronted with numerous challenges in the realm of technology transfer. Investment in research and development (R&D) remains inadequate, while the incentives for innovation are deemed to be inadequate. Moreover, the presence of bureaucratic barriers has been identified as a significant impediment to effective collaboration between institutions and companies. The prevailing culture of collaboration remains underdeveloped (Castro Bermúdez and Murcia González, 2024). Significant lags in innovation have been identified within strategic sectors such as manufacturing and agriculture, which in turn negatively impacts their efficiency and competitiveness (Arangüena Delaude, 2024).

The overcoming of these obstacles is essential to the consolidation of a robust innovation system and the optimisation of technology transfer processes in the country. Technology transfer is of fundamental importance to a nation's economic development and competitiveness, facilitating the transformation of scientific and technological advances generated in academic and research institutions into innovations that are then applied in the productive sector. In Colombia, this process is confronted by numerous challenges that impede the establishment of a connection between knowledge generation and its implementation in industry. Despite the efforts of institutions to strengthen the relationship between academia and the productive sector, there are still significant gaps that limit the effectiveness of these efforts (Galvez Grados and Herrera Quispe, 2020).

One of the primary obstacles pertains to the absence of clearly defined and sustainable policies that would encourage cooperation between universities, research centers, and companies. The absence of adequate incentives for researchers and entrepreneurs has engendered an environment in which technology transfer is not regarded as a strategic priority (Pérez Botero, 2016). Moreover, the complexity of bureaucracy and the intricacies of administrative processes can impede the formalization of collaboration agreements, consequently delaying the implementation of innovations in the market. From an economic perspective, Colombia's comparatively low investment in research and development (R&D) relative to other countries in the region restricts companies' capacity to assimilate and apply novel technologies. It is evident that small and medium-sized enterprises (SMEs) are particularly deficient in the financial and human resources required to adopt technological innovations, thereby creating a significant disparity between scientific knowledge and its practical application. Moreover, the diminution in the budget of the Ministry of Science, Technology, and Innovation (MinCiencias) signifies an added challenge for the development of innovation in the nation (Flores Rueda et al., 2024).

From a cultural perspective, the absence of collaboration between the academic and business sectors has engendered a pervasive mistrust and a reluctance to engage in cooperative endeavors. While companies may perceive academic research as not aligning with their objectives, researchers may harbor reservations regarding the involvement of the private sector in their research endeavors. This disconnection has been identified as a factor hindering the formation of strategic alliances and the implementation of successful technology transfer models (Vargas Nossa, 2021).

Systematic literature reviews are a rigorous and replicable methodology for organizing, evaluating, and synthesizing existing knowledge on a specific topic. They are fundamental to consolidating scientific agendas and supporting evidence-based decision-making. According to Bangdiwala (2024), such reviews facilitate the identification of patterns, knowledge gaps, and levels of evidence in fields characterized by abundant academic production but fragmented research, thereby contributing to the design of more effective public policies and intervention strategies. In contexts such as Colombia, where technology transfer processes encounter structural impediments, a systematic review provides a comprehensive overview and facilitates the establishment of research priorities that are aligned with national needs.

Beyond knowledge synthesis, systematic reviews foster cooperation among researchers, institutions, and productive sectors by providing a common reference framework. Arani et al. (2024) emphasize that these reviews not only consolidate the state of the art but also enable new lines of collaborative research by identifying emerging methodological approaches and areas of opportunity. In the domain of innovation and technology transfer, this instrument integrates dispersed evidence, assesses the effectiveness of implemented policies, and proposes adaptive models tailored to specific territorial and sectoral contexts.

In this vein, the present study employs a systematic review not only as a rigorous methodological approach but also as a means to enhance the articulation between science, technology, and society in Colombia. By underscoring the institutional, economic, and cultural factors that influence technology transfer, this research endeavors to contribute to the formulation of a shared agenda that promotes more inclusive, context-sensitive, and sustainable innovation.

Despite the fact that a number of studies have been conducted on the subject of technology transfer in Colombia, there are still significant gaps in the literature regarding the specific dynamics that limit its effectiveness. The majority of research in this field has been concerned with the innovation system in general terms, rather than exploring the specific structural factors that impede the integration of scientific knowledge into the productive sector. Despite the investment of time and resources by universities and research centers, the results do not always meet expectations due to insufficient funding for innovation and a lack of adequate incentives within academic institutions (Galvez Grados and Herrera Quispe, 2020). It is imperative to undertake a thorough analysis of the prevailing challenges and formulate strategies to address this discrepancy, thereby promoting more efficient and sustainable technology transfer in Colombia.

In this regard, the objective of this research is to analyse the dynamics and challenges affecting technology transfer in Colombia, identifying institutional, economic, and cultural factors that limit its effectiveness and sustainability. In order to achieve this objective, a series of questions are posed in order to analyse the dynamics and challenges affecting technology transfer in Colombia.

1. What institutional barriers hinder collaboration between universities, research centers, and companies?
2. How does investment in research and development influence the effectiveness of technology transfer?
3. How do organizational culture and business perceptions affect technology adoption?
4. What strategies have proven effective in improving technology transfer in other contexts, and how can they be adapted to Colombia?
5. What is the role of government policies in strengthening technology transfer in the country?.

Although previous studies have addressed the topic of technology transfer in Colombia, most have focused on general analyses of innovation systems or on isolated institutional experiences, without offering a comprehensive view of the structural factors that hinder effective knowledge transfer to the productive sector (Donneys González and Blanco Campins, 2016; Rivero et al., 2016). Unlike these fragmented approaches, this study offers a systematic review that integrates institutional, economic, and cultural perspectives, based on the PRISMA 2020 methodology. This allows for a more robust identification of barriers and opportunities, as well as the proposal of differentiated and context-sensitive strategies. By bridging existing knowledge gaps and highlighting the relevance of regional dynamics, this research contributes a more holistic understanding of the challenges and opportunities for strengthening technology transfer in Colombia.

## Methodology

The research employs the PRISMA 2020 methodology (Preferred Reporting Items for Systematic Reviews and Meta-Analyses), an internationally recognized framework that enhances transparency, comprehensiveness, and reproducibility in systematic reviews (Page et al., 2021). This approach provides a rigorous and standardized structure for identifying, selecting, evaluating, and synthesizing relevant scientific studies—particularly valuable in contexts where the available evidence is fragmented, such as technology transfer in countries with diverse institutional and innovation capacities.

The implementation of PRISMA ensures methodological rigor and reduces bias, which is essential for accurately characterizing the institutional, economic, and cultural dynamics that shape technology transfer in Colombia. Furthermore, this protocol enables the comparison of results with studies conducted in other Latin American countries, such as Mexico, Peru, and Argentina, where analogous structural challenges exist, including limited R&D investment, fragmented innovation ecosystems, and weak university–industry collaboration. The increased utilization of PRISMA in the region has resulted in the establishment of a shared methodological foundation for the development of regional research agendas and the promotion of context-sensitive scientific cooperation.

### Eligibility criteria

A range of studies analyzing technology transfer and knowledge exchange in Colombia were included in the review, with a focus on the factors that affect their development and application in the productive sector. The research was selected from studies published in indexed journals, characterized by rigorous methodologies and verifiable results. Documents examining institutional, economic and cultural barriers that limit technology transfer were also included in the study, with a particular emphasis on those utilizing comparative approaches or proposing strategic solutions.

The exclusion process was conducted in three stages. In the first instance, duplicate records or those with metadata inconsistencies, such as incorrect titles, poorly referenced authors, or inadequate database classifications, were eliminated. In the second stage of the selection process, studies that were not available in open access or through institutional subscription or other academic means were discarded. In the third stage of the research process, studies that did not include a specific analysis of technology transfer in Colombia, or which did not meet the criteria of scientific rigor, were excluded from the analysis. Studies employing tangential approaches or which did not make relevant contributions to the analysis objectives were also discarded.

The inclusion criteria considered empirical and theoretical studies published in peer-reviewed and indexed scientific journals that explicitly addressed technology transfer within the Colombian context. Eligible documents included original research articles, systematic or integrative literature reviews, case studies, and analytical essays that met rigorous methodological standards and provided verifiable findings. Studies proposing conceptual models, policy recommendations, or comparative analyses with other Latin American countries were also accepted, provided they included elements applicable to Colombia. Excluded from the review were theses, conference papers, non-peer-reviewed technical reports, and other forms of gray literature that did not meet the standards of scientific rigor.

### Sources of information

The information sources employed in this research comprised the Scopus and Web of Science databases, due to their extensive thematic coverage and recognition within the scientific community. Scopus is a multidisciplinary database that indexes scientific literature from peer-reviewed journals, conference proceedings, and patents. The journal's scope encompasses a wide range of subject areas, providing access to contemporary and pertinent research in the field of technology transfer. Web of Science, conversely, is an indexing platform that compiles high-impact scientific publications and provides tools for analyzing citations and trends in academic production.

The selection of both databases was based on their prestigious reputation, consistent updating, and rigorous indexing of scientific publications. Scopus is distinguished by its extensive collection of indexed documents, while Web of Science is notable for its rigorous selection of high-impact journals. The complementarity of these approaches ensures comprehensive identification of studies on technology transfer in Colombia (Asubiaro et al., 2024).

### Search strategy

The search strategy was structured using specific equations for each database, with the purpose of identifying relevant studies on technology transfer in Colombia. The formulation of these equations was informed by the established inclusion criteria, with a particular emphasis on research that focused on knowledge exchange and its impact on the productive sector.

In Scopus, the following equation was used: (Tl=(“Technology Transfer” OR “Knowledge Transfer”) OR KEY (“Technology Transfer” OR “Knowledge Transfer”)) AND TITLE-ABS-KEY (“Colombia”).

In Web of Science, the syntax was adapted to its search structure: (TS=(“Technology Transfer” OR “Knowledge Transfer”) OR AK=(“Technology Transfer” OR “Knowledge Transfer”)) AND TS=(“Colombia”).

The employment of these equations ensured the exhaustive identification of relevant studies, thereby reducing the risk of omitting key publications and thereby strengthening the validity of the selection process.

To minimize potential bias in the formulation of the search strategy, a careful selection of synonyms and related terms was carried out. Although the core descriptors were “technology transfer” and “knowledge transfer,” the search equations were tested with various keyword combinations and Boolean operators to ensure the inclusion of the most representative studies. The fields TITLE, ABSTRACT, and KEYWORDS were used to capture a broader spectrum of literature and to mitigate omission of relevant documents due to terminological differences across publications.

Furthermore, a double-review process was applied to the screening and selection of results. Two independent reviewers evaluated the search outputs, verified the adequacy of the documents based on inclusion criteria, and resolved discrepancies through discussion and consensus. This method helped reduce selection and interpretation bias, increasing the validity and reliability of the data used in the systematic review.

### Selection process

The selection process was conducted through a peer review process in order to ensure objectivity and rigor in the identification of relevant studies. Initially, a search of equations was conducted in Scopus and Web of Science, yielding a preliminary set of studies.

In the initial phase, records that were duplicates or contained indexing errors were eliminated. A subsequent review of the full-text availability was conducted, resulting in the discarding of inaccessible studies. In the final phase, the relevance and methodological quality of each document were assessed, ensuring their alignment with the inclusion criteria.

**Figure 1:**
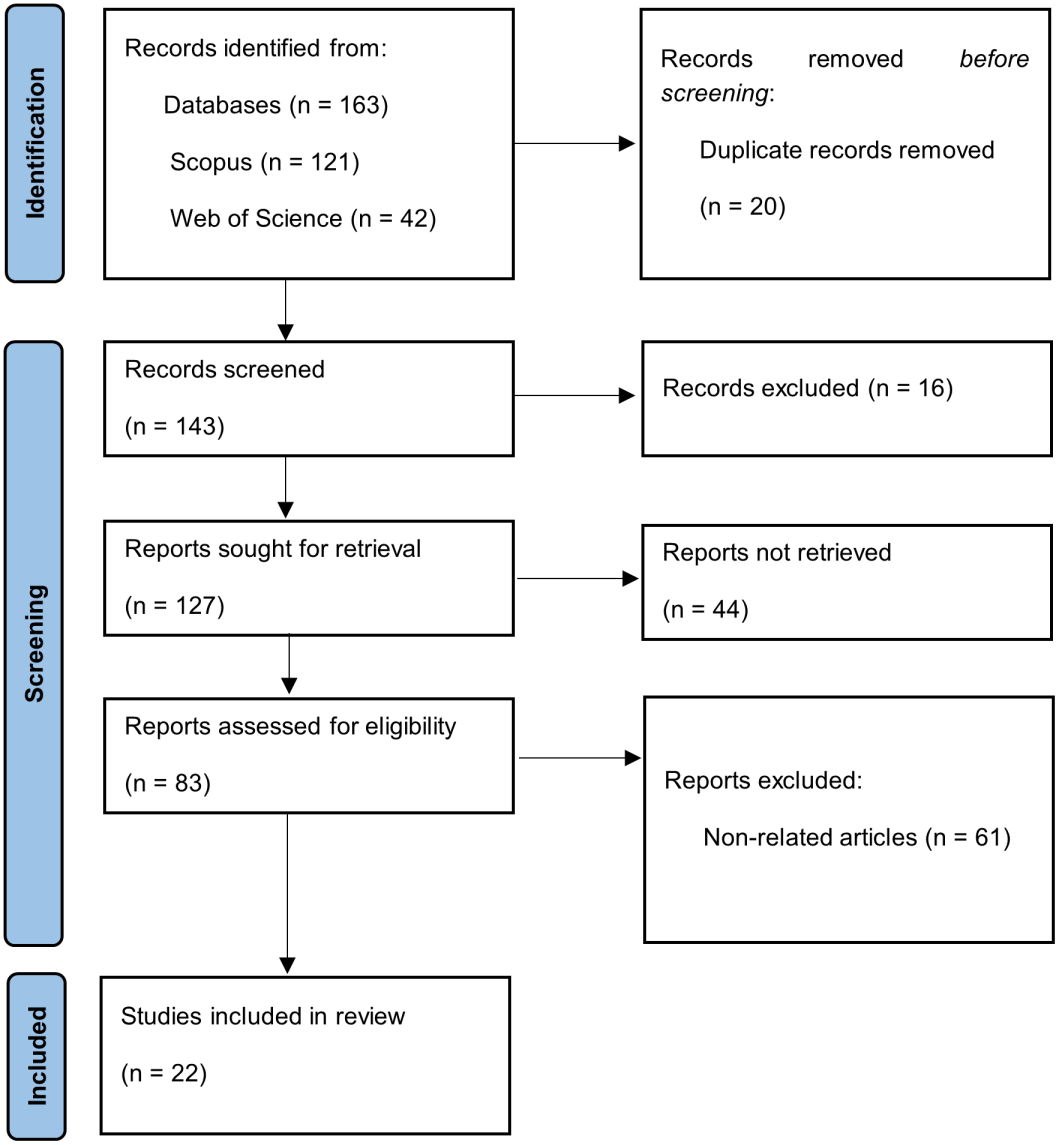
PRISMA flowchart. prepared by the authors based on scopus and web of science.

The final selection was made by consensus among reviewers, with studies that provided clear evidence on the dynamics and challenges of technology transfer in Colombia being prioritized. Figure 1 presents a flowchart based on the PRISMA 2020 declaration, which visualizes the study selection process. The following diagram illustrates the identification, screening, eligibility, and inclusion phases, thereby facilitating a clear and structured representation of the procedure followed in the systematic review.

### Data processing

The data processing was conducted in Microsoft Excel, a software renowned for its ability to organize and analyze information in a structured manner. Initially, data were extracted from the selected studies, with consideration given to title, authors, year, source, methodology, and main findings. The information was then categorized according to the institutional, economic, and cultural factors that affect technology transfer in Colombia. The application of filters and segmentations was undertaken with the objective of facilitating the comparison of studies and the identification of patterns. This process enabled the systematization of the collected evidence and facilitated its analysis based on the research objectives.

### Risk of bias

The potential for bias in this research emerged at various stages of the process, encompassing study selection and analysis. The utilization of databases such as Scopus and Web of Science might have resulted in publication bias, favoring research that has been indexed on these platforms and excluding other potentially relevant sources. The search strategy, predicated on specific search terms, has the potential to limit the identification of studies employing distinct approaches to technology transfer in Colombia. The possibility of reporting bias influencing the results is also a consideration, with some studies omitting negative or less conclusive findings. In order to mitigate the aforementioned risks, a rigorous selection process was implemented, alongside critical analysis of the collected information.

## Results

The results were organized according to the research questions, thus facilitating a structured analysis of the dynamics and challenges affecting technology transfer in Colombia. This structure facilitated the identification of institutional, economic, and cultural factors that were limiting the process, along with the relevant strategies and policies for strengthening them. The findings were constructed from the analysis of specialized literature and selected case studies. Table 1 presents a summary of the studies included in the analysis.

**Table 1:** Studies included in the research.

**Title**	**Authors**
A technology transfer strategy based on the dynamics of the generation of intellectual property in Latin-America	Fuquen and Olaya Escobar, 2018
Industry and academia partnership for aquatic renewable energy development in Colombia: A knowledge-education transfer model from the United Kingdom to Colombia	Colmenares-Quintero et al., 2020
Regional innovation governance and place-based policies: design, implementation and implications	Morisson and Doussineau, 2019
The Adoption of the Latin American Rice Production System through the Implementation of Advanced Field Management Practices: An Evaluation of Technology Adoption Patterns and the Impact on Yield in Colombia	Alwarritzi et al., 2020
The Current Agricultural Technology Transfer Policy in Colombia Modeled through Dynamic Performance Governance	Roa-Ortiz and Cala-Vitery, 2025
A look at the evolution of the academic spin-offs creation in iberoamerican countries: The cases of spain and colombia; [Una mirada a la evolución de la creación de spin-offs académicas en países iberoamericanos: Los casos de españa y colombia]	Morales Gualdrón, 2020
A Machine Learning Model for the Diagnosis of Coffee Diseases	Martinez et al., 2022
A way forward on adaptation to climate change in Colombian agriculture: Perspectives toward 2050	Ramirez-Villegas et al., 2012
Acquisition, transfer and utilization of knowledge in vallecaucanas SMES (Colombia); [Adquisición, transferencia y utilización del conocimiento en las PYMES Vallecaucanas (Colombia)]	Riascos-Erazo et al., 2025
Analysis of the problems derived from COVID-19 and its effects on agricultural transformation processes: the experience of the ReaCTÍvate Santander program; [Análisis de las problemáticas derivadas de la COVID-19 y sus efectos en los procesos de transformación agrícola: la experiencia del programa ReaCTÍvate Santander]	Jaimes et al., 2024
Botswanan palm basketry among the Wounaan of western Colombia: Lessons from an intercontinental technology transfer	Bernal et al., 2013
Center for Water Research: Leading edge technology wins international projects	Imberger et al., 2007
Compatibility of Ancestral and Innovative Agricultural Practices in the Kankuamo People of Colombia; [Compatibilidade de práticas agrícolas ancestrais e inovadoras no povo Kankuamo da Colômb0ia]; [Compatibilidad de prácticas agropecuarias ancestrales e innovadoras en el pueblo Kankuamo de Colomb0ia]	Rivera et al., 2021
Distance learning: introducing Felix Candela's concrete shells in Cali	Botti, 2019
Genetically Modified Organisms as Public Goods: Plant Biotechnology Transfer in Colombia	Holmes and Graham, 2009
Guidelines for a methodology to identify learning styles suitable for the Colombian agricultural sector; [Lineamientos para una metodología de identificación de estilos de aprendizaje aplicables al sector agropecuario colombiano]	Rodríguez-Espinosa et al., 2020
Intermediation for technology diffusion and user innovation in a developing rural economy: a social learning perspective	Theodorakopoulos et al., 2014
Knowledge Management and its impact on Social Performance in Solidarity Organizations: The role of Absorptive Capacity and Organizational Learning	Silva et al., 2024
Knowledge management in the south colombian fish culture sector: A strategy of technological transfer; [Gestión del conocimiento en el sector piscícola surcolombiano: Una estrategia de transferencia tecnológica]	Andrade et al., 2018
Teaching locals new tricks: Foreign experts as a channel of knowledge transfers	Markusen and Trofimenko, 2009
Transferring technology from university to rural industry within a developing economy context: The case for nurturing communities of practice	Theodorakopoulos et al., 2012
University objectives and socioeconomic results: A multicriteria measuring of alignment	Cortés-Aldana et al., 2009

Prepared by the authors based on Scopus and Web of Science.

**Figure 2:**
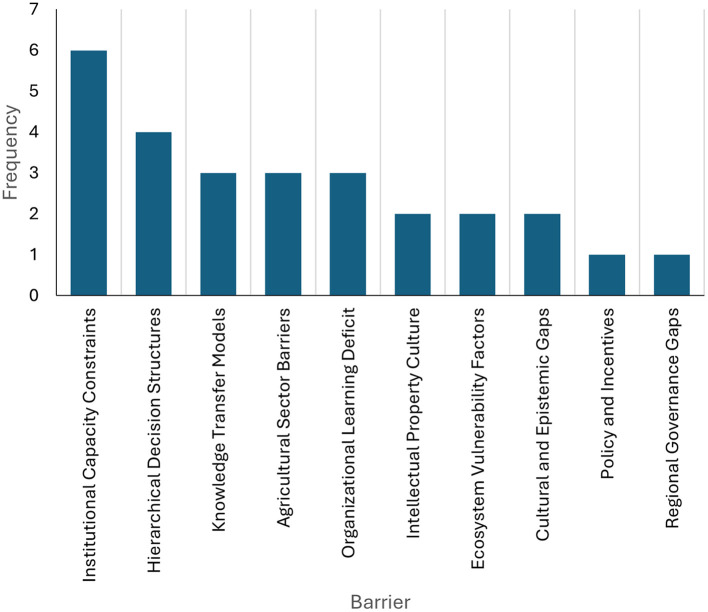
Most reported institutional barriers. Prepared by the authors based on Scopus and Web of Science.

As illustrated in Figure 2, the primary institutional impediments impeding university-business collaboration in technology transfer processes in Colombia are evident. The text highlights institutional capacity limitations with six mentions, followed by hierarchical decision-making structures (Arangüena Delaude, 2024), knowledge transfer models (Castro Bermúdez and Murcia González, 2024), agricultural sector barriers (Castro Bermúdez and Murcia González, 2024), and organizational learning deficits (Castro Bermúdez and Murcia González, 2024). Other barriers include intellectual property culture, system vulnerability factors, cultural and epistemic gaps, as well as aspects related to policies, incentives, and regional governance, each with a lower frequency.

**Figure 3:**
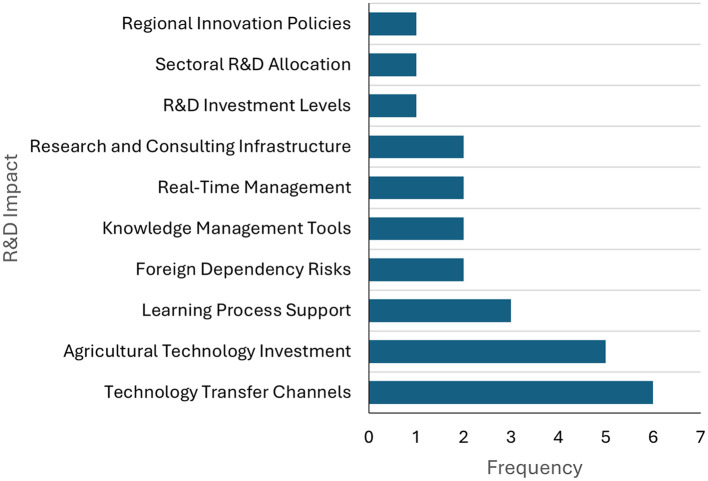
Key aspects of R&D investment. Prepared by the authors based on Scopus and Web of Science.

As illustrated in Figure 3, the primary aspects associated with investment in research and development (R&D) and its repercussions on technology transfer in Colombia are outlined. The most frequently referenced subjects are technology transfer channels (six mentions) and investment in agricultural technology (five). It is evident that the following elements are of particular significance in this context: firstly, the support for learning processes (Castro Bermúdez and Murcia González, 2024); secondly, the risks of foreign dependence (Rivero et al., 2016); thirdly, the knowledge management tools (Rivero et al., 2016); fourthly, the real-time management (Rivero et al., 2016); fifthly, the infrastructure for research and consulting (Rivero et al., 2016); and sixthly, the individual mentions of R&D investment levels, sector allocation, and regional innovation policies.

**Figure 4:**
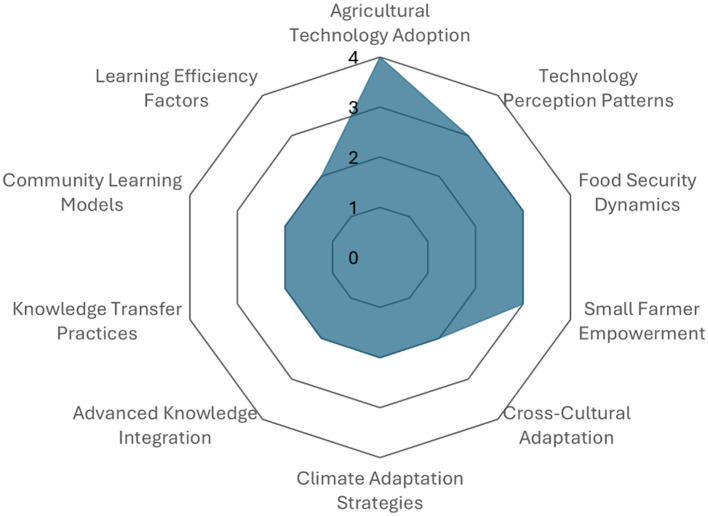
Organizational culture and technological adoption. Prepared by the authors based on Scopus and Web of Science.

As illustrated in Figure 4, the primary components associated with organizational culture within the context of technology perception and adoption are delineated. The study's key findings are as follows: firstly, the adoption of agricultural technology; secondly, technology perception patterns; thirdly, food security dynamics; and finally, the empowerment of small farmers. The following factors were also identified: cross-cultural adaptation, climate change strategies, integration of advanced knowledge, knowledge transfer practices, community learning models, and learning efficiency.

**Figure 5:**
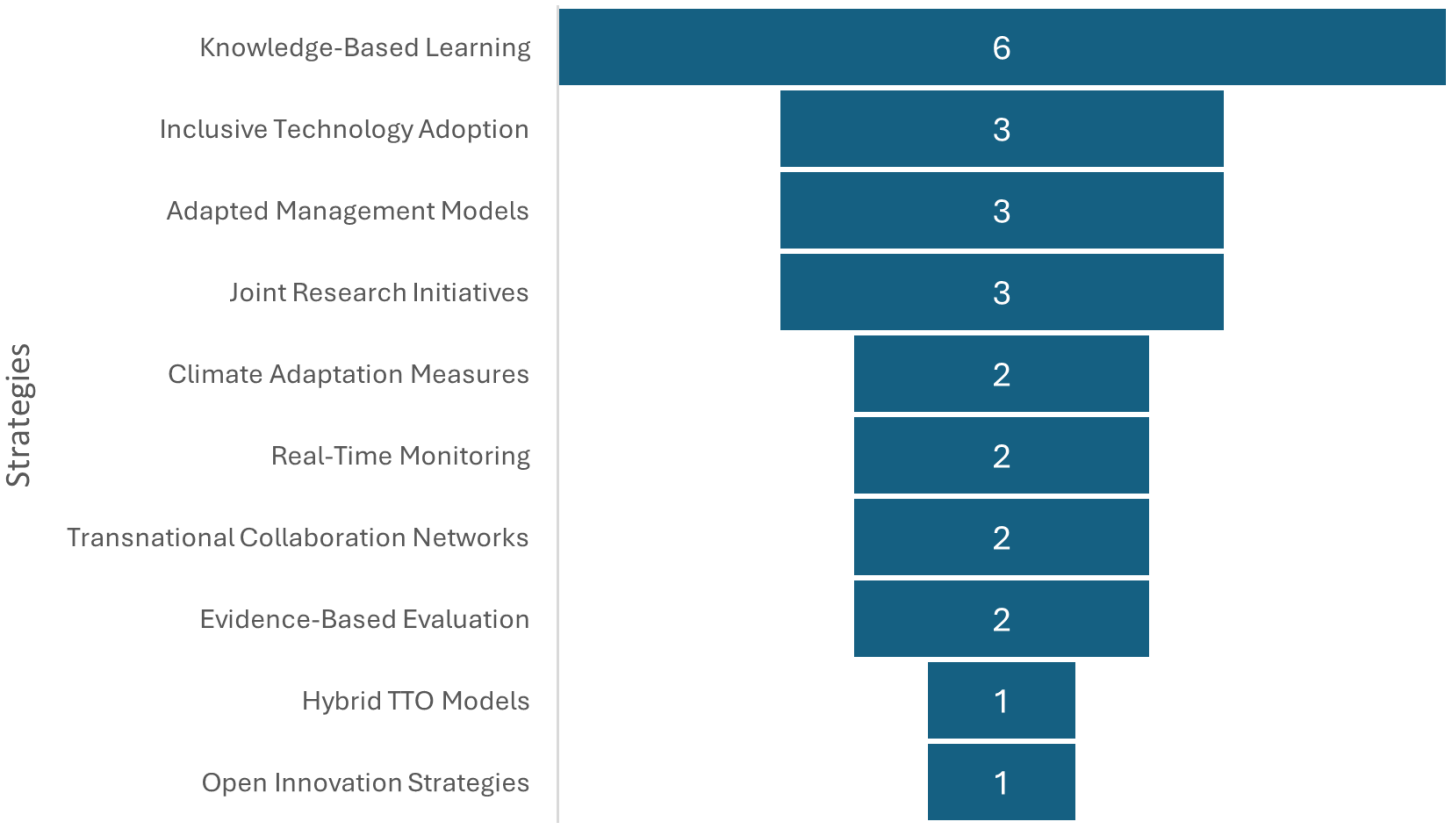
International strategies for technology transfer. Prepared by the authors based on Scopus and Web of Science.

Figure 5 identifies effective strategies derived from international experiences that can be adapted to the Colombian context in order to strengthen technology transfer. The findings of the study include knowledge-based learning, inclusive technology adoption, and adapted management models. A plethora of additional features have been identified, including, but not limited to, joint research initiatives, climate adaptation measures, real-time monitoring, transnational collaboration networks, evidence-based assessment, as well as hybrid technology transfer office (TTO) models and open innovation strategies.

**Figure 6:**
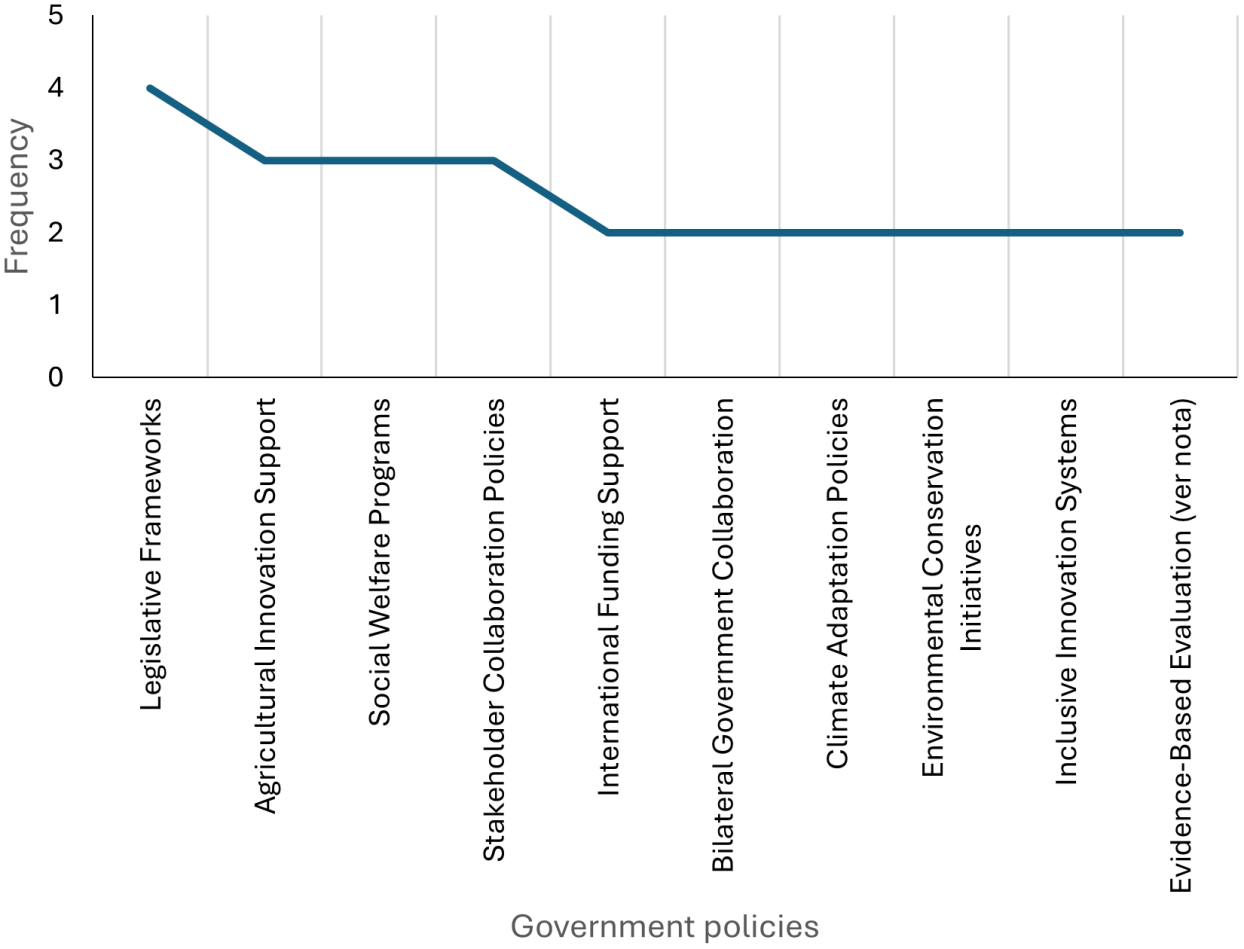
Public policies to strengthen technology. Prepared by the authors based on Scopus and Web of Science.

As illustrated in Figure 6, the systematic review has identified the primary components of government policies that have contributed to technological strengthening in Colombia. The results of the study highlight the importance of legislative frameworks, support for agricultural innovation, and social welfare programs. In addition, policies for collaborative action, international financing, bilateral cooperation, climate change adaptation, environmental conservation initiatives, inclusive innovation systems, and evidence-based assessment mechanisms are identified, reflecting a comprehensive approach to the formulation of public policies aimed at technology transfer.

## Discussion

This section discusses the findings of the study on the dynamics and challenges of technology transfer in Colombia. The main patterns identified are interpreted based on the theoretical framework and the national context. The results of the present study are then compared with those of previous studies conducted in Colombia and other countries, with a view to highlighting both similarities and differences between them, and identifying any gaps in the research. The results obtained have enabled the formulation of a conceptual framework, the purpose of which is to summarize the critical factors of the process. The study's theoretical, policy, and practical implications are also presented, its limitations are identified, and future research is proposed.

### Analysis of results

As demonstrated in Figure 2, the findings reveal that institutional barriers persist as a substantial impediment to technology transfer processes in Colombia, particularly within university-business collaborations. The dissemination of knowledge is constrained by factors such as capacity limitations, hierarchical structures, and a paucity of a culture of intellectual property. As Holmes and Graham (2009) have observed, researchers operating within Colombia encounter challenges pertaining to intellectual property rights and regulatory restrictions. In a similar vein, Theodorakopoulos et al. (2014) emphasize that technology intermediation processes necessitate the surmounting of institutional deficiencies to ensure effective transfer in rural economies and underdeveloped sectors.

As demonstrated in Figure 3, the results indicate a close correlation between R&D investment in Colombia and technology transfer channels, as well as strategic sectors such as agriculture. This demonstrates a focus on strengthening applied capabilities, although challenges associated with external technological dependence and fragmented knowledge management persist. As posited by Holmes and Graham (2009), Colombian researchers encounter substantial impediments to the advancement of biotechnology, largely attributable to financial constraints and regulatory frameworks. In a similar vein, Navia et al. (2018) identify a weak local appropriation of knowledge, with a strong inclination toward acquiring it from external contexts.

The results presented in Figure 4 demonstrate that organizational culture exerts a direct influence on technology adoption in Colombia, particularly within agricultural contexts. The following factors have been identified as being key to facilitating transfer: technological perception, food security, and the empowerment of small producers. Additionally, elements such as cross-cultural adaptation, community learning, and the integration of advanced knowledge are emphasized. These findings are consistent with the study by Zambrano Dueñas and Zambrano Zambrano (2023), which demonstrates how the adoption of technologies such as mechanized planting and the utilization of certified seeds enhances yield in rice systems. Furthermore, the present study aligns with the arguments posited by Martinez et al. (2022) concerning the role of institutional culture in fortifying the transfer system through the establishment of academic spin-offs.

As demonstrated in Figure 5, the findings indicate that one of the primary obstacles to technology transfer in Colombia is the absence of pedagogical strategies that have been adapted to the rural context. The adoption of low-technology is associated with the absence of methodologies that consider producers' learning styles. Moreover, within indigenous communities such as the Kankuamos, tensions have been identified between ancestral practices and modern technologies, necessitating diversified approaches to technology adoption (Pérez Botero, 2016). The two studies under consideration both highlight the necessity for culturally relevant training processes in order to improve the effectiveness of technology implementation.

The process of technology transfer in Colombia is characterized by the need to reconcile traditional practices with modern innovations. In communities such as the Kankuamos, technology adoption necessitates the implementation of diversified approaches that take into account sociocultural and environmental factors (Rivera et al., 2021). Conversely, technologically advanced sectors such as rice farming demonstrate that the coordinated implementation of technological packages can significantly enhance productivity when adapted to local conditions (Alwarritzi et al., 2020). The analysis of both cases indicates that the efficacy of technology transfer is contingent on the implementation of context-specific strategies, emphasizing a participatory approach and ensuring congruence between ancestral knowledge and scientific advances.

### Comparison of results with other studies

The findings from research on technology transfer in Colombia illustrate the necessity for a diversified strategy that takes into account sociocultural factors, particularly in rural or indigenous contexts, such as that of the Kankuama community. This outcome is consistent with the findings of a study conducted by Sanchez Preciado (2018), who analyzed transfer processes in rural areas of Cauca. The findings of both studies indicate that these processes necessitate knowledge adaptation and constant interaction between the actors involved. Furthermore, consensus has been reached that transfer should not be unidirectional or based on universal frameworks, but rather contextual and collaborative. From an institutional perspective, Fuquen and Olaya Escobar (2018) posit that technology transfer offices in Latin America have been implemented without taking into consideration the specificities of each region. The findings of this study corroborate this assertion by demonstrating that adoption strategies must be adapted to local conditions, such as the prior exclusion of agrochemicals. The findings of both studies indicate the necessity of establishing alternative mechanisms that do not depend exclusively on patents, but rather integrate traditional knowledge with scientific innovation.

Bejarano et al. (2023) posit that the efficacy of technology transfer within academic institutions is enhanced by open innovation, a concept that fosters collaboration with regional stakeholders within the innovation system. The findings of this research lend support to this hypothesis by highlighting the role of entities such as AGROSAVIA in co-creation processes with communities. This articulation demonstrates a clear commitment to the adoption of sustainable technologies, thereby fostering a robust nexus between the university, the local community, and the productive sector. Conversely, Correa et al. (2014) have highlighted that the modifications introduced to Colombia's science and technology policy have not yielded the desired enhancements in terms of the quality or the impact of the transfer process. Whilst the present research does not directly assess the aforementioned indicators, it does identify structural gaps, such as those related to health and education, that hinder technology appropriation. This finding indicates that effective technology policies must be aligned with social policies.

The study by Burbano-Vallejo (2024) concludes that the focus of Colombian literature on academic spin-offs is more oriented toward their creation than on their sustainability. Despite the shift in focus from technology generation to its actual impact, the research indicates a discrepancy between the two. The experience with biofortification in vulnerable communities demonstrates that technological success requires social relevance and long-term sustainability. The findings of this study are consistent with the most recent literature in indicating that technology transfer should be oriented toward comprehensive, user-centered models that are sensitive to local contexts. These results support the need for a review of traditional models and a shift toward more collaborative and adaptive approaches.

### Proposed conceptual framework

**Figure 7:**
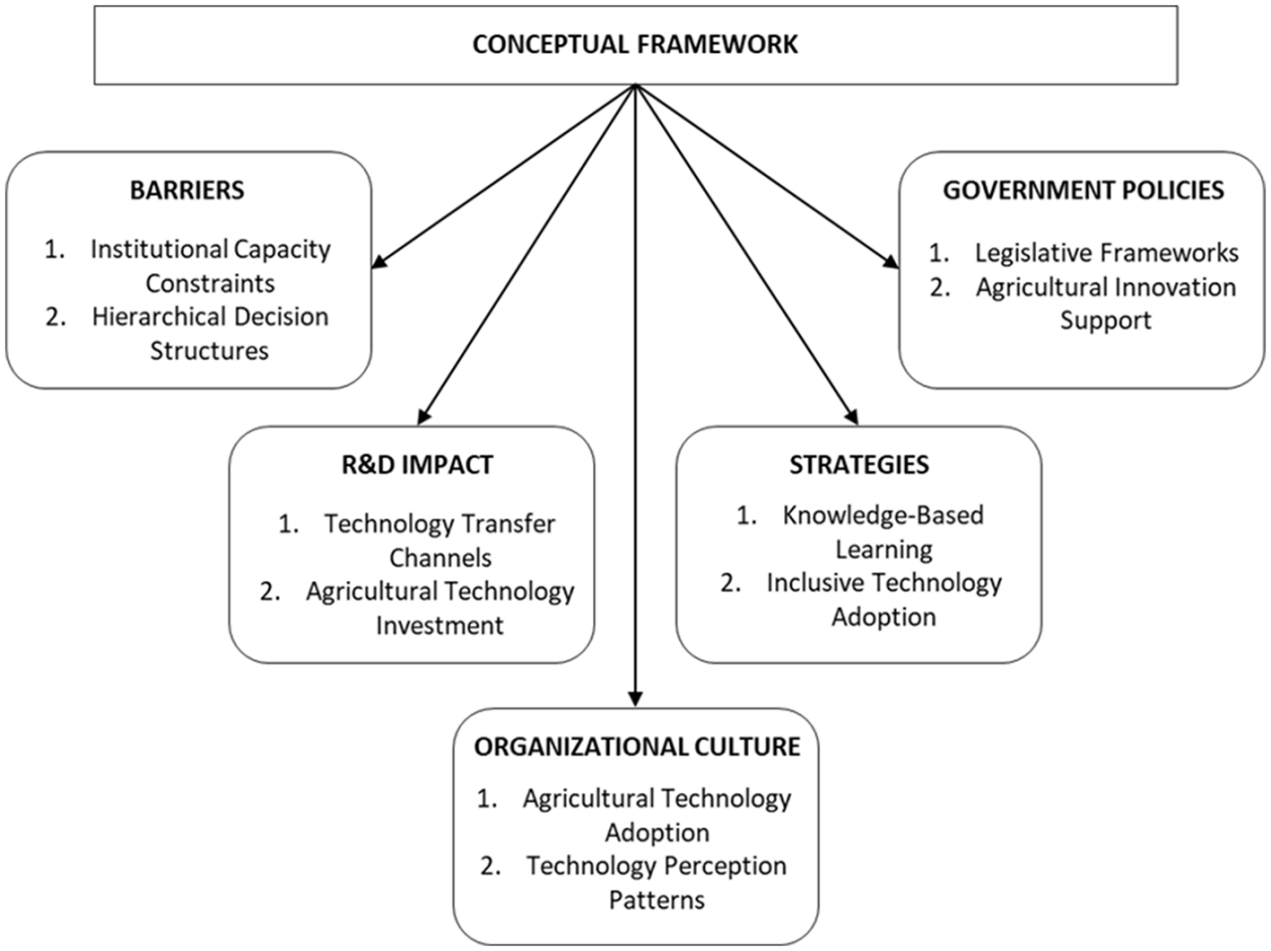
Conceptual framework for technology transfer in Colombia. Prepared by the authors.

As illustrated in Figure 7, the integration of the primary findings is facilitated by a conceptual framework that organizes institutional barriers, R&D investment challenges, organizational culture, government policies, and effective strategies derived from international benchmarking. The model employs a systems-based approach to illustrate technology transfer in Colombia as a network of interconnected actors, institutional levels, and territories. The report incorporates the impact on productivity as a cross-cutting axis and proposes paths to strengthen local capacities, improve university-business-community coordination, and promote context-adapted innovation.

The proposed conceptual framework gains robustness when compared with international evidence on technology transfer challenges. In the United Kingdom, Vick and Robertson (2018) conducted a systematic literature review highlighting that university–industry collaboration is often hindered by a disconnect between academic motivations and the actual needs of industry. Their study emphasizes the importance of addressing sociopolitical and contextual variables simultaneously—an aspect our model integrates by considering not only institutional structures, but also cultural and territorial dynamics that affect technology adoption.

In the context of China, Guo et al. (2024) developed a system dynamics model to evaluate the implementation effectiveness of technology transfer policies. Their work demonstrated the value of modeling feedback loops between research, transfer, and industrialization stages, and how policy tools can intervene at critical points to improve outcomes. This insight supports the systemic orientation of our framework, which incorporates policy adaptability and institutional coordination as key levers for improving transfer processes in Colombia.

Italy offers another relevant reference point, especially in regions with traditional or low-technology industries. Grimaldi et al. (2021) analyzed how Italian universities have developed multifaceted roles in response to regional economic contexts. Their findings emphasize the need for differentiated strategies that acknowledge local production structures and dynamics. This aligns with our model's territorial focus, which advocates for region-specific innovation strategies and a stronger connection between universities, local businesses, and communities.

### Implications

The findings of this research make a significant contribution to the existing literature on technology transfer from a contextual perspective. The extant evidence demonstrates the manner in which institutional, economic, cultural, and territorial dynamics in Colombia affect the circulation of knowledge. In principle, this necessitates a transition from linear models to systemic approaches. This implies the recognition of three factors. Firstly, the diversity of actors must be recognized. Secondly, the asynchrony of capabilities must be recognized. Thirdly, the relevance of local knowledge must be recognized. The inadequate articulation between universities, businesses, and communities is indicative of historical, cultural, and epistemic structures that influence the generation, validation, and application of technology.

This multi-scale analysis serves to expand the conceptual framework for countries in the Global South by incorporating variables such as territorial governance, risk perception, and the integration of ancestral knowledge. Investment in R&D is not regarded as an isolated factor, but rather as a component linked to institutional frameworks and mechanisms for technological appropriation. This provides a foundation for new research directions, specifically investigating the impact of innovation policies in contexts characterized by regional inequalities.

At the political level, the results indicate the need for flexible regulatory frameworks with a territorial focus. The disconnection between technological and social policies has been demonstrated to impede transfer processes, particularly within sectors such as agriculture. It is recommended that public policies be formulated with a view to acknowledging local conditions, encouraging collaboration between the public, private and academic sectors, and fostering adaptive governance. It is imperative that these policies offer differentiated incentives and establish evaluation systems that measure social and environmental impacts.

It is imperative to enhance transfer offices through technical training, continuous financing, and collaborative networks. The adoption of strategies such as open innovation and co-creation has been demonstrated to facilitate environments of trust and collaboration. The integration of R&D investment with local productive development plans has been demonstrated to be a successful strategy for narrowing economic disparities, generating employment opportunities, and enhancing territorial competitiveness.

In practical terms, the findings offer guidelines for universities, companies, innovation centers, and transfer offices. It is recommended that universities undertake a review of their knowledge management models with a view to incorporating social impact criteria. It is imperative that academic production is linked to local solutions and social innovation processes. Researchers' competencies in transfer issues require enhancement, as do institutional incentives to foster intersectoral collaboration. It is imperative for businesses, particularly those of a small and medium size, to be provided with technical and financial support so that they can identify relevant technologies, adapt solutions, and connect with innovation networks. It is imperative that technology transfer offices assume a mediating role, with the capacity to effectively coordinate interests and timelines across different sectors. In order to achieve this objective, it is necessary to employ participatory methodologies, to utilize technological intelligence, and to engage in continuous knowledge management. It is imperative that innovation centers consolidate their position as articulation hubs that promote the exchange of experiences, access to shared infrastructure, and training in innovation management. It is imperative that collaboration between these actors is predicated on a shared conception of technology transfer as a reciprocal process. In this process, knowledge is mobilized, transformed, and applied with regional relevance.

This study makes a significant contribution to the existing body of knowledge on technology transfer in Colombia. The proposal sets out a pragmatic programme of action intended to surmount obstacles, galvanize local capabilities, and establish an inclusive, robust innovation system that is attuned to the specific circumstances of the region.

## Limitations

This study on the dynamics and challenges of technology transfer in Colombia presents methodological limitations that must be acknowledged to strengthen its scientific soundness. The qualitative approach facilitated detailed exploration; however, it limits the possibility of extrapolating the results to other settings. The data collection process encompassed interviews and a comprehensive documentary analysis. However, it is important to acknowledge the inherent subjectivity of this analysis, which is contingent on the disposition, context, and interpretation of the participants.

This can result in the introduction of biases associated with individual experiences or institutional structures. The analysis concentrated on representative cases, without providing a comprehensive overview of all regions or sectors. The absence of quantitative data impeded the establishment of direct relationships between variables such as investment, productivity, or social impact. Technology transfer is a dynamic process; therefore, the findings should be understood as an approximation situated in a specific time and place. The identification of these limitations enables the development of new research avenues that complement, deepen, and contrast the results obtained.

One important limitation of this study concerns the formulation of the search equation used in the systematic review. The query included only English-language terms such as “technology transfer” and “knowledge transfer,” excluding potentially relevant results that could have been retrieved using equivalent Spanish terms such as “innovación” or “colaboración universidad-empresa.” Additionally, the absence of a defined temporal window may have affected the scope of the analysis, particularly given the growth in indexed publications on Colombia since 2005. Expanding the search to include Spanish-language terms and a time-based filter would likely yield a broader corpus of studies; however, such changes would require a complete restructuring of the methodology and were therefore acknowledged as a methodological limitation rather than implemented in the present study.

## Lines of future research

The results, implications, and limitations identified in this study allow us to propose new lines of research to further investigate technology transfer in Colombia. A priority line of enquiry involves the undertaking of comparative studies between regions exhibiting varying degrees of institutional development, research and development capacities, and levels of coordination between universities, businesses, and communities. This approach facilitates the identification of differential factors that explain variability in transfer processes and allows us to extract useful lessons for contexts with less maturity in innovation.

Furthermore, there is a necessity to advance longitudinal studies that analyze the evolution of inter-institutional links, the effects of sustained investment in science and technology, and the cumulative results of implemented public policies. This perspective facilitates the overcoming of a static view and the observation of processes of learning, adaptation, and institutional transformation. Another relevant line of research proposes the use of mixed methodologies that integrate qualitative and quantitative data. The proposal entails the conceptualization of specific indicators to assess the effectiveness of technology transfer offices, the social impact of adopted innovations, and the return on investment in collaborative projects. These tools facilitate the establishment of relationships between variables such as investment, productivity, technological appropriation and territorial sustainability.

In addition, further investigation is required into the processes of cultural, epistemic, and organizational mediation that influence transfer. This analysis encompasses the examination of the negotiation of meanings, the adaptation of technologies, and the establishment of relationships of trust between actors with divergent logics. It is recommended that the integration of traditional knowledge and scientific knowledge, as well as the co-creation models present in rural and peri-urban contexts, be explored in greater detail. In conclusion, the proposal is made for the study to be undertaken of the development of institutional and human capacities that are necessary for effective transfer.

This field encompasses the analysis of training practices, organizational learning mechanisms, and collaborative leadership strategies within universities, companies, and innovation centers. Addressing these dimensions enables a more comprehensive and situated approach, consistent with the country's structural and territorial challenges. These research lines serve to consolidate the academic field, whilst concurrently proffering pertinent elements for the design of public policies and knowledge-based development strategies.

## Conclusion

The phenomenon of technology transfer in Colombia must be considered as a socio-political process, rather than a technical process of knowledge circulation. It is a complex, dynamic, and systemic phenomenon subject to various variables that impede its progress in the country, such as: (a) institutional barriers; (b) a lack of investment in R&D&I; (c) organizational culture and business perception; (d) a lack of implementation of international strategies applicable to the context; (e) a limited scope of government policies that streamline these processes. It is important to note that all of the aforementioned factors are also influenced by institutional structures, territorial inequality, and cultural diversity.

The study posits that institutional barriers stem from a paucity of collaboration among stakeholders within the innovation system, attributable to organizational rigidity and an absence of technology transfer models that are adapted to the realities of the context. Moreover, the persistent underfunding of R&D&I in the country has been shown to impede the establishment of effective channels for technology transfer and investment in this domain, thereby hindering the adoption of novel technologies.

With regard to organizational culture, the results indicate that it plays a leading role in the adoption of technologies, which in turn drives technology transfer. This variable reveals a disconnection between the technologies offered by academia and the needs of businesses. This constitutes a challenge that must be addressed to strengthen ties and ensure that the business community sees universities as a source of technological solutions adapted to the context.

In addition, there are international success stories, as well as various strategies that could incorporate context and strengthen technology transfer: knowledge-based learning, inclusive technology adoption and adapted management models, as well as joint research initiatives, climate adaptation measures, real-time monitoring, transnational collaboration networks, evidence-based evaluation, and finally, hybrid technology transfer office (TTO) models and open innovation strategies.

It is therefore unsurprising that public policies are deemed to be of crucial importance for the strengthening of technology transfer in the country. In order to facilitate the streamlining of these processes, it is essential to establish comprehensive legislative frameworks that can support, encourage, and incentivize engagement between stakeholders. In emerging countries, these have been shown to be key factors in improving the innovation environment through inclusion policies, international financing, and bilateral cooperation, which will help reduce structural barriers and foster a more integrated innovation system.

In conclusion, it is evident that technology transfer in Colombia necessitates a comprehensive approach that simultaneously addresses institutional, economic, and cultural factors. It is imperative to strengthen existing public policies, increase investment in R&D, foster a culture of cooperation between academia and the business sector, and learn from successful international experiences to design strategies tailored to local needs. In order to achieve the optimization of technology transfer and, by extension, the economic development and competitiveness of the nation, it is essential that there is a greater degree of collaboration and alignment of interests among the key stakeholders.

The analysis demonstrates that, in order to comprehensively address the relationship between science, technology and society, it is imperative to undertake a systematic review of the conceptual frameworks that guide this relationship. National evidence indicates that imported, decontextualized, or linear models reproduce forms of exclusion when they ignore local trajectories, community dynamics, and ancestral knowledge.

Achieving effective transfer requires dialogue between diverse rationalities, ongoing adaptation processes, and recognition of the scales at which innovation is produced and appropriated. In order to move forward with this endeavor, it is essential to build trust among stakeholders, to understand the sociocultural environments in which they operate, and to strengthen institutional capacities. These conditions permit the transformation of conventional approaches and the generation of new forms of interaction.

A review of these dimensions has been shown to enhance academic comprehension of the phenomenon, thereby providing essential components for the formulation of inclusive and sustainable policies. The promotion of technology transfer with a territorial focus and social commitment is not merely an operational challenge. This decision is indicative of a conscientious approach to the nation's equitable development.

## Recommendations

In response to the challenges affecting the success of technology transfer in Colombia, the study proposes six strategic lines of action aimed at strengthening and enhancing these processes across different levels and sectors. The first strategy emphasizes the importance of flexible policy formulation and capacity building, highlighting that public policies must be able to adapt to evolving circumstances and promote collaboration among stakeholders in the national innovation system. These policies should not remain confined to the national level but must also be deployed across regional and local governance structures. In particular, it is essential to support the creation, revitalization, and long-term sustainability of Technology Transfer Offices (OTRIs) by equipping them with the necessary resources, personnel, and tools to fulfill their central role in technology transfer and capacity building. Equally important is the simplification of administrative procedures between system actors, especially in formalizing agreements, establishing conventions, and facilitating the creation of academic spin-offs.

The second strategic line calls for the design of regional innovation policies tailored to the specific characteristics and needs of local productive sectors. These policies must address critical areas such as agricultural innovation, environmental sustainability, and climate change adaptation. Furthermore, it is crucial to strengthen international cooperation through research networks and collaborative projects, and to develop targeted support mechanisms for SMEs—considered the backbone of Colombia's productive system—through training, financing, and network integration, thus facilitating their entry into the national innovation ecosystem.

A third priority involves increasing investment in research, development, and innovation (R&D&I). While Colombia has some fiscal and financial incentives in place, their impact remains limited unless they are continuously promoted and refined. There is a clear need for the creation of more programs, projects, and policy instruments that stimulate innovation, along with mechanisms that foster public-private partnerships to support technology transfer, particularly in strategic sectors of the economy.

The fourth line of action underscores the necessity of building a culture of trust, collaboration, and cooperation among key stakeholders. Effective technology transfer depends on strong relationships between academia, industry, and other actors, and it is therefore essential to promote engagement activities that foster mutual trust and highlight successful case studies. Academic institutions play a central role here by developing initiatives that help other stakeholders recognize the value of technology transfer and its contributions to innovation.

The fifth strategy focuses on adapting international models for technology transfer to the Colombian context. While it is useful to study and implement practices from established Technology Transfer Offices abroad, these models must be adjusted to reflect local realities and leverage domestic knowledge to strengthen national strategic sectors. To do so, it is necessary to build meaningful relationships with international peers, networks, and programs that facilitate knowledge exchange and collaboration, ultimately enhancing Colombia's ability to integrate into the global innovation system.

Finally, the sixth strategic line addresses the imperative of aligning technology transfer with sustainability and climate change adaptation. Technological development cannot be detached from environmental and social concerns, particularly in rural and marginalized communities. The technologies promoted must address real community needs and prioritize sustainability goals, including efficient energy use, sustainable agriculture, and climate resilience. A key element is the incorporation of communal models that integrate ancestral, Afro-Colombian, indigenous, and local knowledge with new technologies, ensuring inclusive and context-sensitive innovation processes that promote both environmental stewardship and social equity.

## Data Availability

The raw data supporting the conclusions of this article will be made available by the authors, without undue reservation.
